# From Algorithm Performance to Laboratory Deployment: A Critical Review of Artificial Intelligence in Clinical Chemistry

**DOI:** 10.3390/molecules31203617

**Published:** 2026-10-11

**Authors:** Abdelraheem Baniahmad, Sulieman Bani-Ahmad

**Affiliations:** 1 Department of Medical Analysis, Faculty of Applied Medical Sciences, Al al-Bayt University, Mafraq 25113, Jordan; 2 Department of Intelligent Systems, Faculty of Artificial Intelligence, Al-Balqa Applied University, Salt 19117, Jordan; sulieman@bau.edu.jo

**Keywords:** clinical chemistry, laboratory medicine, artificial intelligence, machine learning, large language models, nanobiosensors, analytical validation

## Abstract

Artificial intelligence (AI) is increasingly being applied in clinical laboratories to support laboratory processes and interpretation of test results. Recent developments in machine learning, deep learning, and large language models have expanded the potential applications of AI across different stages of laboratory testing. However, the use of AI in clinical laboratories also raises important questions regarding validation, analytical performance, and transferability between laboratories. This narrative review examines recent applications of AI across the total testing process in clinical chemistry. Recent English-language scientific literature published from 2023 to 18 August 2026 was identified through searches of PubMed, Scopus, and Google Scholar. It describes AI-assisted test utilization; specimen handling and quality assessment; detection of laboratory errors; patient-based quality control; interpretation of complex analytical outputs, including signals from emerging AI-integrated biosensors and nanomaterial-based analytical platforms; result verification; and laboratory-report interpretation. The review also discusses major implementation challenges, including analytical variability, data quality, sensor and platform reproducibility, model generalizability, interoperability, and limitations of large language models. Particular attention is given to external validation, transportability, and real-world implementation. Among the studies considered in this review, many applications remain supported mainly by retrospective or internally validated studies, while prospective and operational evaluations are less common. Finally, practical considerations for validation, local verification, quality assurance, and performance monitoring are discussed. AI has considerable potential to support clinical chemistry laboratories, but safe use requires appropriate evaluation and continued involvement of laboratory professionals.

## 1. Introduction

Clinical chemistry laboratories are highly automated environments that generate large volumes of longitudinal and interconnected data in structured, semi-structured, and unstructured formats. Modern laboratory information systems (LISs), middleware, total laboratory automation, and electronic health records provide an infrastructure in which artificial intelligence (AI) can be incorporated throughout the testing process. Machine learning (ML), deep learning, computer vision, natural language processing, and, more recently, large language models (LLMs) have broadened the capabilities of laboratory automation beyond conventional rule-based systems. Unlike traditional approaches that rely primarily on predefined criteria and fixed decision rules, these AI-based methods can learn complex patterns from data and support tasks such as prediction, classification, image interpretation, text analysis, and clinical decision support [1,2,3,4].

Recent AI applications span many components of laboratory practice. ML models have been developed to improve test utilization, identify specimen misidentification and contamination, support patient-based quality control, recognize patterns in electrophoresis and urinalysis, estimate reference intervals, and assist result verification [3,4,5,6,7,8]. Recent reviews and surveys also describe growing interest in generative AI for natural-language interaction, laboratory knowledge support, and interpretation-related tasks [1,3,9]. The rapid expansion of the field is reflected in recent reviews of laboratory ML and in professional recommendations from the International Federation of Clinical Chemistry and Laboratory Medicine (IFCC) and the European Federation of Clinical Chemistry and Laboratory Medicine (EFLM) [2,3,4,5,6,7,8].

Clinical chemistry is well suited to AI applications because it generates large volumes of predominantly quantitative data that are routinely stored in LISs and are standardized to a considerable extent. However, laboratory-specific analytical and preanalytical factors can influence the data supplied to AI models and may therefore affect model transportability across laboratories [5,6,7,8]. Consequently, an AI model developed and validated in one laboratory may not achieve the same performance when applied in another laboratory. This variability highlights the importance of external validation across different laboratory settings and analytical platforms before clinical implementation.

Emerging analytical technologies are also broadening the types of laboratory signals that can be combined with AI. Biosensors incorporating functionalized nanomaterials can generate highly sensitive optical, electrochemical, plasmonic, or spectroscopic readouts, while ML can assist with signal processing, feature extraction, pattern classification, and quantitative interpretation [10,11]. Although these platforms remain largely translational rather than routine in clinical chemistry, they illustrate how AI may become integrated not only with laboratory information systems but also directly with next-generation analytical devices.

**Figure 1:**
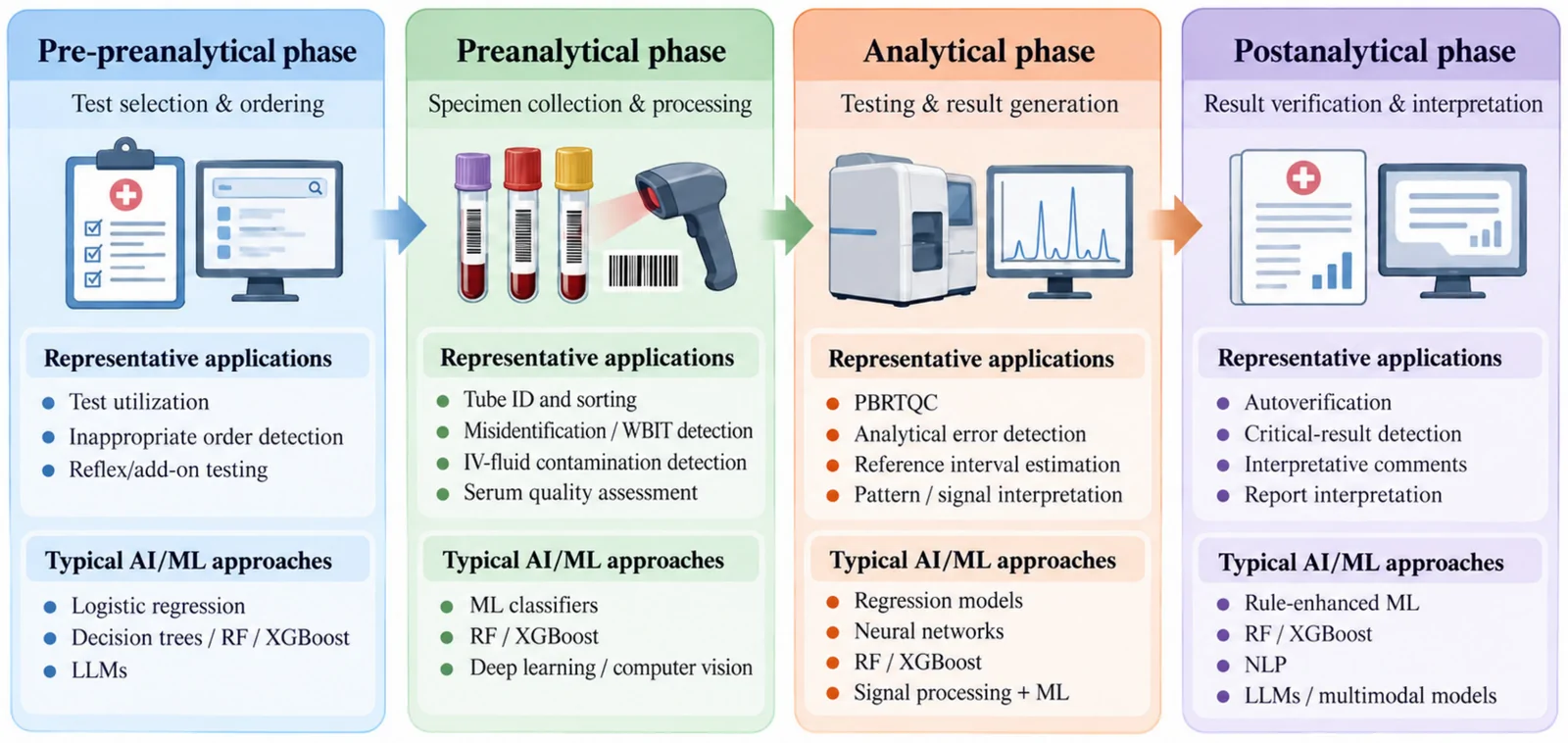
AI applications across the total testing process in clinical chemistry. Representative applications are shown for the pre-preanalytical, preanalytical, analytical, and postanalytical phases, together with typical AI/ML approaches used within each phase.

AI applications do not always align neatly with the traditional phases of the total testing process. In this review, applications are classified primarily according to the laboratory process or source of error they address rather than the point at which the algorithm technically operates. Test selection is therefore treated as a pre-preanalytical activity; specimen collection, identification, and quality problems as preanalytical; measurement quality and patient-based monitoring as analytical; and verification, reporting, and interpretation as postanalytical (Figure 1). Reference-interval estimation and interpretation of analyzer-generated patterns are discussed with analytical applications because of their close relationship to analytical data generation, although they may be regarded as postanalytical activities in some laboratory workflows.

This narrative review examines recent AI applications across the pre-preanalytical, preanalytical, analytical, and postanalytical components of clinical chemistry practice. The primary focus is clinical chemistry; selected studies from other areas of laboratory medicine are included when they illustrate principles directly transferable to clinical chemistry, such as specimen misidentification, multimodal image interpretation, or post-deployment monitoring. The review summarizes current applications, compares the evidence supporting them, and discusses analytical and operational considerations relevant to routine implementation. This article also considers analytical variability, external validation, real-world deployment, AI-integrated nanobiosensors, LLM-specific challenges, and lifecycle issues across the total testing process.

## 2. Literature Search and Study Selection

A structured literature search was conducted using PubMed, Scopus, and Google Scholar to identify relevant English-language publications from 2023 to 18 August 2026. Search terms were used in different combinations and included: artificial intelligence, machine learning, deep learning, large language models, clinical chemistry, laboratory medicine, clinical laboratory, preanalytical, analytical, postanalytical, quality control, autoverification, specimen identification, reference intervals, electrophoresis, urinalysis, and biosensors. Reference lists of relevant reviews and key articles were also examined to identify additional studies. The 2023–2026 time window applied primarily to the recent scientific literature; earlier foundational, regulatory, normative, and standards documents were included when relevant to the conceptual, regulatory, or quality-management aspects of the review.

The review focused primarily on AI applications in clinical chemistry. Studies from other areas of laboratory medicine were included when they addressed principles directly relevant to clinical chemistry, such as specimen misidentification, error detection, analytical quality monitoring, multimodal laboratory-data interpretation, or post-deployment monitoring. Original studies were prioritized for discussion of model development, validation, performance, and implementation, while reviews, professional recommendations, and regulatory or normative documents were used to provide broader context. Studies were selected based on their relevance to laboratory practice, methodological importance, and contribution to understanding validation, generalizability, implementation, or clinical workflow. Publications with limited relevance to these objectives were not prioritized for detailed discussion.

Generative AI was used for language editing and for assistance in the preparation and editing of graphical material; it was not used for study selection, data extraction, or determination of the scientific conclusions. All scientific content, numerical data, and references were reviewed and verified by the authors.

## 3. Conceptual Framework for AI in Clinical Chemistry

**Figure 2:**
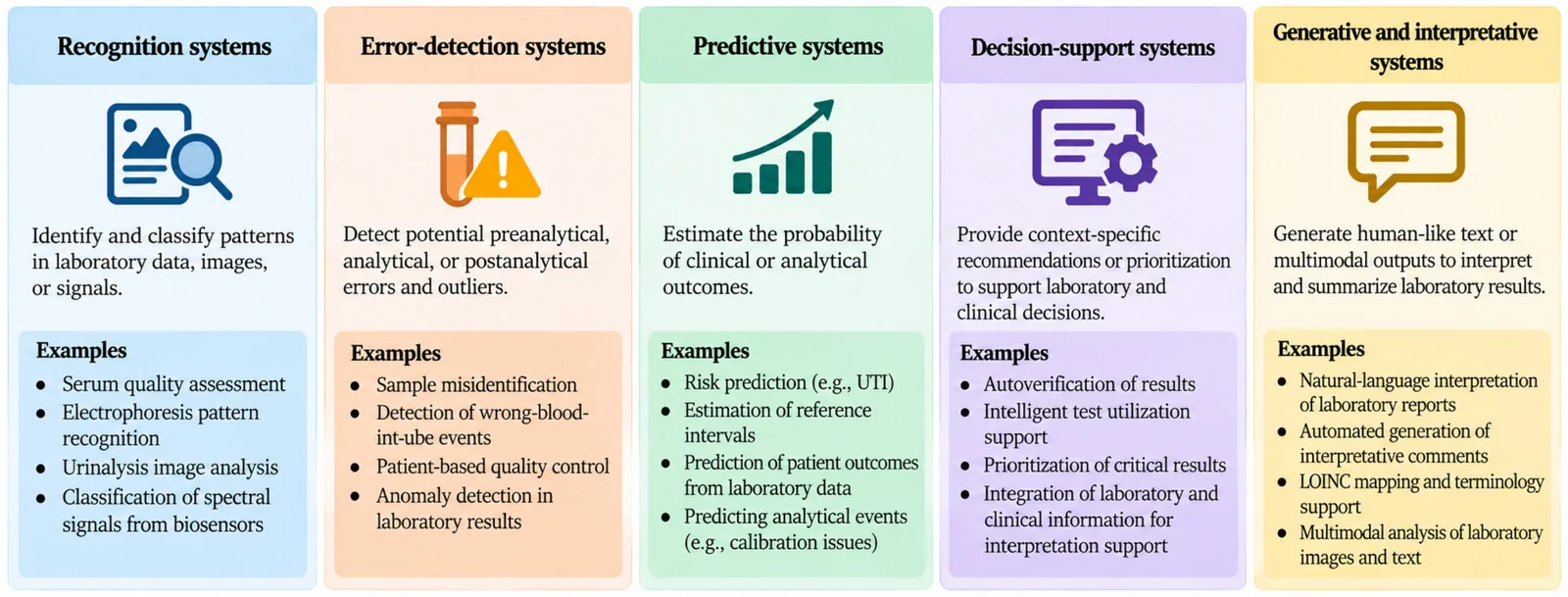
Functional taxonomy of AI applications in clinical chemistry. Representative applications are organized according to their principal function as recognition, error-detection, predictive, decision-support, and generative or interpretative systems. Abbreviations: WBIT, wrong-blood-in-tube; PBRTQC, patient-based real-time quality control; UTI, urinary tract infection; LOINC, Logical Observation Identifiers Names and Codes.

AI applications in clinical chemistry may vary substantially in their targeted function, complexity, and potential impact on patient care. For the purposes of this review, these applications are organized into five functional categories: recognition, error detection, prediction, decision support, and generative or interpretative systems (Figure 2). This classification provides a practical framework for comparing heterogeneous AI applications according to their principal function within the laboratory process while recognizing that individual systems may incorporate features from more than one category.

### 3.1. Recognition Systems

Recognition systems are designed to identify intrinsic patterns within laboratory data (tabular data or images) without necessarily generating clinical predictions. Examples may include serum-quality assessment, urinalysis image classification, and electrophoretic pattern recognition [12,13,14,15,16,17,18].

### 3.2. Error-Detection Systems

Error-detection systems aim to identify probable deviations from expected laboratory processes, including specimen misidentification, contamination, wrong-blood-in-tube (WBIT) events [19,20,21,22,23,24,25,26], and analytical errors detected through patient-based real-time quality control (PBRTQC) [27,28,29,30,31,32,33,34].

### 3.3. Predictive Systems

Predictive systems use ML models to predict analytical or clinical outcomes based on previous laboratory data. An example is the prediction of sepsis using laboratory measurements [35].

### 3.4. Decision-Support Systems

Decision-support systems assist laboratory or clinical decision-making by supporting test utilization, laboratory result verification, autoverification, or diagnostic assessment [36,37,38].

### 3.5. Generative and Interpretative Systems

Generative and interpretative systems include LLMs and multimodal models capable of producing natural-language explanations, interpretative comments, recommendations, or clinical knowledge support [39,40,41,42,43,44,45,46,47,48,49,50,51,52,53,54].

The functional categories described here represent distinct modes of AI use within the laboratory rather than a linear continuum. Their clinical significance and validation requirements are determined by the context in which they are applied, particularly the intended use.

## 4. Recent AI Applications in the Pre-Preanalytical and Preanalytical Phases

The pre-preanalytical and preanalytical phases offer several useful targets for AI because they include high-volume decision making, large numbers of specimens, repetitive tasks, and recognizable error patterns. Recent applications include support for test selection, tube identification and sorting, specimen quality assessment, detection of WBIT events, and recognition of contamination [12,19,20,21,22,23,24,25,26,36,37,55,56,57].

AI-assisted test utilization is an early application of AI in the laboratory testing pathway. Computational models can support appropriate test selection, reduce unnecessary or repeated testing, and guide clinicians toward more efficient use of laboratory resources. Yang et al. evaluated the generalizability of an ML model designed to improve utilization of parathyroid hormone-related peptide testing across multiple clinical centers [36]. More recently, Zayed et al. evaluated LLMs as laboratory-test recommenders in primary- and emergency-care scenarios [37]. These studies suggest that computational decision-support systems may complement laboratory stewardship by incorporating previous laboratory results and relevant clinical information. Evaluation should not depend only on agreement with expert recommendations; studies should also assess inappropriate recommendations, unnecessary additional testing, clinician rejection of recommendations, and possible diagnostic delay.

AI has also been introduced into specimen reception and processing. Şişman et al. evaluated an AI-based robotic system designed to sort blood tubes and detect preanalytical errors. In a preliminary local validation, 1000 blood tubes were evaluated under seven predefined scenarios and tested in five repeated runs, yielding 5000 sorting and error-detection instances. The system achieved a reported overall accuracy of 99.98%, detected all tubes containing predefined preanalytical errors, and processed approximately 311 tubes per hour [56]. Because the evaluation used an experimental test set with predefined error scenarios rather than the natural prevalence of errors during routine operation, these figures should not be interpreted as evidence of equivalent performance in unrestricted clinical use. Demirci later evaluated another AI-supported system for blood-tube labeling and handling under operational conditions [57].

AI has also been evaluated for serum-quality assessment. Chen et al. studied a two-stage deep-learning approach for automated recognition of hemolysis, icterus, and lipemia. A primary dataset of 21,000 serum samples was divided into training and validation sets, and a separate independent test dataset of 21,000 samples was used for final evaluation. In the independent test dataset, the model achieved binary classification accuracies of 97% for hemolysis, 93% for icterus, and 97% for lipemia [12]. Such systems may be particularly useful where specimen acceptance still depends partly on visual inspection. Because visual grading is subjective and may differ among observers, automated or AI-assisted assessment could improve consistency and reproducibility of specimen quality evaluation.

Detection of specimen misidentification is one of the most widely studied preanalytical applications. Conventional delta checks compare current and previous results to identify possible patient or specimen mismatch. ML can extend this approach by evaluating several analytes and their relationships simultaneously. Seok et al. developed ML-based delta checks for tumor markers using 246,261 records and found that deep neural networks provided more stable discrimination of simulated misidentification than conventional delta percent change, absolute delta percent change, and reference-change-value methods [19]. Farrell et al. also demonstrated ML-based detection of WBIT using complete blood count (CBC) data [20].

Seok et al. later examined sample-misidentification detection using 397,751 results for training and internal validation and 215,339 results from three independent laboratories for external validation. Deep neural networks and extreme gradient boosting (XGBoost) models performed better than conventional approaches, although performance differed among tumor markers and external datasets [21]. Graham et al. extended multianalyte WBIT detection to a predominantly pediatric population and demonstrated an important practical limitation: high overall accuracy does not necessarily produce a high positive predictive value when the event being detected is rare. When evaluated under a simulated low-prevalence setting, estimated positive predictive value (PPV) varied markedly with the selected threshold, ranging from 0.01 to 0.67 for CBC with differential and from 0.01 to 0.75 for CBC without differential [22].

Sürmeli et al. examined analyte importance in WBIT detection using almost 800,000 samples and reported that small combinations of CBC indices, particularly mean corpuscular volume (MCV) and red cell distribution width (RDW), maintained strong discriminatory performance [23]. Tortosa-Carreres et al. subsequently reported development and external validation of ML-based WBIT detection in hospitalized patients [24]. Together, these studies show progression from proof-of-concept models toward broader validation while also emphasizing the importance of prevalence and threshold selection.

Intravenous (IV) fluid contamination is another area in which relationships among several laboratory results may be informative. Spies et al. evaluated GPT-4 for recognition of simulated IV-fluid contamination and found that trained healthcare professionals performed better than the LLM [25], illustrating the limitations of a general-purpose generative model for a specialized laboratory task. The same group later developed an unsupervised ML approach using a very large dataset of basic metabolic panels. The model identified patterns consistent with contamination and detected cases that had not been recognized during routine laboratory practice [26].

Farrell also applied multivariate anomaly detection to routine clinical chemistry results and showed that relationships among analytes can help identify unusual result combinations that may not exceed conventional limits for any single analyte [58]. Such methods may be useful for detecting contamination, specimen mix-up, and other errors because abnormal relationships among several measurements can reveal patterns that are difficult to detect using individual results alone.

Overall, studies addressing pre-preanalytical and preanalytical problems show a shift from fixed rule-based screening toward systems that evaluate multiple analytes, historical patterns, or specimen images. The strongest evidence comes from clearly defined laboratory problems with measurable outcomes and direct comparison against established approaches such as expert specimen inspection, conventional delta checks, or current laboratory procedures. A recent critical review likewise summarizes the growing use of ML for laboratory error detection and highlights barriers to routine implementation [59].

## 5. Recent AI Applications in the Analytical Phase

This section considers AI applications related to analytical quality as well as data-driven use of analyzer-generated outputs and routine laboratory databases. Current research focuses mainly on PBRTQC, detection of analytical errors, estimation of reference intervals, and interpretation of complex analytical patterns [13,14,15,16,17,18,27,28,29,30,31,32,33,34,35,60,61,62,63,64,65,66,67,68,69].

PBRTQC uses the continuous flow of patient results to identify changes in analytical performance. Traditional methods usually rely on moving averages, moving medians, or similar statistical approaches and can support conventional internal quality control by monitoring performance between scheduled control measurements. However, their performance can be affected by changes in the patient population. AI-based methods have therefore been developed to consider more complex patterns in patient data and improve the detection of small analytical shifts [27,28,29,30,31,32,33,34].

Liang et al. developed nonlinear ML-based PBRTQC models for 10 measurands using data from eight hospitals and evaluated the models in three independent hospitals [27]. The multicenter design addresses differences in patient populations and testing practices among institutions. The ML approach improved detection of simulated analytical bias compared with conventional PBRTQC methods, particularly for some measurands and smaller analytical errors.

Dong et al. compared AI-supported PBRTQC with traditional PBRTQC using six biochemical and immunological analytes [28]. They evaluated error detection, false-positive and false-negative rates, the number of patient samples required before an error was detected, and overall discrimination. In the evaluated conditions, the AI-assisted approach more efficiently identified several quality risks and, for selected analytes and parameter settings, required fewer patient results for detection.

Additional approaches have also been studied. Shang et al. combined anomaly detection and graph neural networks with patient and laboratory information for sodium, potassium, and calcium. Their system reduced the number of patient samples required to detect simulated analytical errors compared with conventional PBRTQC methods, particularly for some small biases [29]. Duan et al. reviewed recent developments in PBRTQC and highlighted the increasing use of ML and multivariable approaches [30], while Liu et al. developed a patient-based quality-control method using a voting algorithm [31].

Xia et al. combined neural-network prediction with joint probability analysis. Patient demographic information was used to predict expected laboratory values, and differences between predicted and measured values were then monitored. At the same target false-alarm rate, the neural-network method required fewer patient results to detect analytical errors than traditional PBRTQC in the evaluated datasets [32]. Zhou et al. proposed another neural-network-based PBRTQC approach [33]. More recently, Su et al. compared neural-network, pre-classified, and traditional PBRTQC methods for six tumor markers. At a target false-alarm rate of 0.1%, the neural-network method reduced the trimmed number of patient results affected before error detection by an average of 62% compared with traditional PBRTQC [34].

Collectively, these studies indicate that AI-based approaches can improve the sensitivity or flexibility of patient-based analytical monitoring under selected conditions. PBRTQC nevertheless remains sensitive to changes in the clinical population. Hospital activity, patient age, referral patterns, season, the proportion of inpatients and outpatients, and changes in test-ordering practices can alter patient-result distributions. AI may account for some of these patterns, but it may also learn laboratory-specific structure. Multicenter validation and careful control of false alarms are therefore essential.

Analytical quality is also important because laboratory measurements are frequently used as inputs to AI models. Although not an ML study, Andersen et al. examined analytical performance specifications for variables included in the Model for End-Stage Liver Disease and demonstrated the broader principle that errors in individual measurements can propagate into multivariable clinical outputs [60]. The same principle is relevant to ML models that combine multiple, often correlated, laboratory variables.

Yesil et al. examined this issue directly in a support vector machine model for sepsis prediction. They introduced 26 combinations of analytically plausible biases into laboratory measurements. Model performance changed to varying degrees across the bias scenarios, although differences in area under the receiver operating characteristic curve were not statistically significant in the evaluated comparisons [35]. The study nevertheless illustrates why ML models should be challenged with realistic combinations of analytical bias rather than assuming that acceptable performance of each individual assay guarantees stability of the combined model.

Measurement uncertainty, method-specific bias, reagent-lot changes, calibration differences, and analyzer replacement should therefore be considered when AI systems are validated [35,60,61]. One practical approach is analytical stress testing, in which model performance is reassessed after introducing realistic changes such as bias, imprecision, missing data, or altered analytical methods. Scenario-based quality control of complex algorithms has also been proposed to apply established laboratory quality principles to AI outputs [62].

Experience with implemented AI systems also shows that the algorithm itself can influence future data. Day et al. described an AI label-feedback loop during retraining of a clinically implemented system [63]. Once the AI model had influenced human classifications, those decisions became part of the dataset used for later retraining and affected the apparent performance of the updated model. This issue is relevant to laboratory systems that learn from previously verified results. Independent datasets reviewed by humans may therefore be needed to evaluate later versions of such models objectively.

AI can also extract additional information from routine laboratory databases. Reference-interval estimation is one example because laboratories often have large retrospective datasets but may lack sufficient carefully selected reference individuals for every age, sex, and analytical subgroup. Klawitter et al. used regression-tree modeling to automate age- and sex-specific partitioning of reference intervals. The resulting partitions were generally similar to established pediatric partitions, although clinically important differences remained for some analytes [64]. Their findings also emphasize that automatically generated partitions require expert review because statistically separable groups are not necessarily clinically meaningful groups.

Schär et al. included diagnostic information from International Classification of Diseases, 10th Revision (ICD-10) coding in indirect reference-interval estimation, allowing laboratory data to be refined according to associated health status [65]. Böhm et al. later evaluated automated age- and sex-based partitioning using routine laboratory data [66]. Kayahan et al. extended this approach using ML and fuzzy clustering for thyroid-function tests. From 48,397 laboratory records, 9455 individuals were selected as a reference sample, and fuzzy clustering was used to produce continuous rather than sharply separated age-related reference limits [67]. Reviews of newer reference-interval methods suggest that continuous and data-driven approaches may increasingly complement traditional methods [68].

The main value of these approaches is their ability to make better use of routine laboratory data while accounting for gradual physiological changes such as aging. However, their use still requires careful assessment of population selection, possible inclusion of individuals with disease, analytical methods, measurement units, and stability over time.

AI has also been evaluated where laboratory outputs contain complex visual or multidimensional patterns. Serum protein electrophoresis (SPE) and immunofixation electrophoresis (IFE) are important examples. Hu et al. trained an explainable deep-learning system using more than 12,000 expert-annotated IFE images and reported expert-level recognition of several immunoglobulin and light-chain patterns [13]. The system also highlighted image regions contributing to its classifications, allowing laboratory professionals to review the basis of the output.

Elfert et al. evaluated five ML methods for M-protein detection using data from 69,722 individuals. On an independent test set of 1000 samples, their random-forest model achieved 99.1% accuracy, 89.9% sensitivity, 99.8% specificity, an F1 score of 93.2%, a PPV of 96.9%, and a negative predictive value (NPV) of 99.3%, with performance that compared favorably with participating laboratory experts [14]. Lahtiharju et al. later reported retrospective validation of AI-assisted SPE interpretation in 34,625 Finnish patients, supporting the possibility of partial automation in an independent population [15].

More recent studies have combined different types of laboratory information. Zhao et al. combined electrophoretic findings with other laboratory measurements in a deep-learning model for immunoglobulin typing [16]. Oztas and Kosesoy applied transfer learning to 5226 IFE images and reported high accuracy for both binary classification and subclassification tasks [17]. These studies suggest a possible future role for AI in performing high-volume screening, recognizing straightforward normal and monoclonal patterns, and directing uncertain cases to laboratory specialists for review.

Urinalysis provides another example because it generates numerical, image-based, and instrument-derived information. De Bruyne et al. reviewed the increasing use of AI for recognition and interpretation of urine particles [18]. Favresse et al. later combined demographic information with dipstick reflectance measurements in ML models for predicting urinary tract infection (UTI). The study included 22,961 samples and reported values for areas under the receiver operating characteristic curve (AUROC) of approximately 0.92–0.95, suggesting potential value for laboratory screening and prioritization of samples for urine culture [69]. These studies illustrate how information already produced by laboratory analyzers can be repurposed to support additional laboratory decisions.

## 6. AI-Integrated Nanobiosensors and Functionalized Nanomaterials

Nanobiosensors represent an emerging interface between analytical chemistry, functional nanomaterials, and computational interpretation. Metallic nanoparticles such as gold and silver nanoparticles, together with magnetic nanoparticles, quantum dots, carbon-based nanomaterials, and hybrid nanocomposites, can be incorporated into sensing platforms to increase surface area, improve target capture, amplify optical or electrochemical signals, or enable multiplexed detection. Surface functionalization with antibodies, aptamers, nucleic-acid probes, enzymes, or other recognition elements provides molecular selectivity, while AI and ML can be used to denoise complex signals, extract informative features, classify multidimensional response patterns, and convert sensor outputs into quantitative or categorical results [10,11]. These approaches are particularly relevant to point-of-care testing and to analytes present at concentrations that challenge conventional readout strategies.

Recent studies illustrate how nanomaterial-based sensing and ML can be combined for clinically relevant measurements. Dixon et al. coupled silver nanoparticle-based surface-enhanced Raman spectroscopy (SERS) with ML to classify simulated serum samples containing combinations of cardiovascular disease biomarkers while also showing that experimental design and spectral-acquisition conditions can materially influence classifier performance [70]. Wang et al. integrated a gold nanoparticle-based colorimetric lateral-flow assay with ML for quantitative detection of the Alzheimer disease biomarker tau and demonstrated the approach in human plasma [71]. In clinical serum, Zhu et al. combined a Au/SnO2 nanostructured SERS substrate with ML for discrimination of healthy controls, colorectal precancerous lesions, and colorectal cancer, reporting an area under the receiver operating characteristic curve of 0.96 [72]. Ditta et al. similarly used gold nanoparticle-based SERS and ML for classification of serum samples from individuals with and without diabetes [73]. Collectively, these examples show how AI can extract diagnostically useful information from complex signals generated by nanostructured analytical platforms rather than functioning only as a downstream interpreter of conventional laboratory results.

Clinical translation of these systems creates a two-layer validation problem because both the analytical platform and the computational model can contribute to error. Variability in nanoparticle synthesis, surface functionalization, receptor density, nanomaterial aggregation, matrix effects, nonspecific adsorption, fouling, calibration, and signal drift, as well as differences among reagent or fabrication batches, can alter the data supplied to the model [10,70]. Validation should therefore assess conventional analytical characteristics such as precision, detection capability, selectivity, interference, linearity where applicable, and between-batch reproducibility together with model calibration, robustness, and external validation. Independent evaluation across fabrication batches, instruments, specimen matrices, and clinical populations is particularly important when an algorithm may learn platform-specific signal characteristics. In this setting, computational sophistication cannot compensate for inadequate chemical selectivity or unstable analytical performance; the sensor and the AI model should be evaluated as components of a single analytical system. Overall, these studies represent predominantly proof-of-concept or translational applications, and evidence of routine clinical laboratory deployment remains limited.

## 7. Recent AI Applications in the Postanalytical Phase

The postanalytical phase has developed rapidly because clinical laboratories must review, release, contextualize, and communicate large numbers of results. Conventional autoverification applies predefined limits, delta rules, analytical flags, and logical relationships. ML can integrate larger numbers of variables and historical patterns, while generative AI adds natural-language interaction and interpretation [38,39,40,41,42,43,44,45,46,47,48,49,50,51,52,53,54].

Zhu et al. developed an AI-assisted verification system for a clinical biochemistry laboratory using 6382 laboratory reports containing 38 biochemical variables and demographic information [38]. Three models were evaluated using five-fold cross-validation. Random forest showed the highest performance, with an AUROC of 0.985 and an F1 score of 0.91, followed by XGBoost (AUROC 0.970; F1 score 0.87), whereas logistic regression showed substantially lower discrimination (AUROC 0.742; F1 score 0.59). The superior performance of the tree-based ensemble models in this setting may reflect their ability to capture nonlinear relationships and interactions among multiple laboratory variables, whereas logistic regression offers greater simplicity and interpretability but is less flexible for complex multivariable patterns. In double-blind operational testing, the system achieved a pass rate of 88.14% with a false-negative rate of 0.62%, while median turnaround time decreased from 89 to 68 min. Thus, the study demonstrated not only differences in predictive performance among algorithms but also a measurable effect on laboratory workflow.

For verification applications, the consequences of different errors should be reported separately. An unnecessary hold delays a valid result and increases staff workload; an inappropriate automatic release may allow a clinically or analytically problematic result to reach the clinician. Future studies should therefore report false-release and false-hold rates, manual-review burden, handling of critical results, turnaround time, and performance in the subsets of results that laboratory specialists consider difficult.

Generative AI has rapidly expanded the range of postanalytical applications investigated in laboratory medicine. Yang et al. described the foundations and potential roles of AI chatbots in clinical laboratory medicine shortly after widespread public availability of modern LLMs [39]. The EFLM Working Group on AI subsequently evaluated the potential and limitations of ChatGPT for understanding laboratory results [40], while Munoz-Zuluaga et al. examined the accuracy and clinical utility of ChatGPT in laboratory medicine [41]. These early studies established a pattern that has persisted: LLM performance depends strongly on the specific task, input format, model version, and available context.

Abusoglu et al. examined chatbot performance across 60 questions involving preanalytical, analytical, and postanalytical laboratory errors [42]. ChatGPT-4 and CopyAI substantially outperformed ChatGPT-3.5 in several components, demonstrating that model choice and version can materially affect laboratory problem-solving performance. Girton et al. compared ChatGPT with medical professionals for responses to laboratory-medicine questions posted on social media [43], while He et al. evaluated generative models against peer-user answers for explaining laboratory-test results to lay users [44]. Meyer et al. compared ChatGPT, Gemini, and Le Chat with physician interpretations of laboratory questions from an online health forum [45]. Collectively, these studies show useful explanatory capability but persistent variability in completeness, contextualization, and clinical appropriateness.

D’Urso et al. provide an example of domain-restricted laboratory interpretation [46]. The investigators evaluated a Claude-based system using 100 laboratory reports from three Italian laboratories employing different analytical platforms and reporting standards. Three experienced laboratory-medicine reviewers evaluated the output. Within this controlled study environment, the authors reported 100% technical interpretation accuracy and no hallucinations; patient evaluation included 70 participants, 90% of whom provided positive ratings. The model was deliberately instructed to interpret laboratory data without providing a clinical diagnosis.

The study also illustrates the influence of model selection and prompt control. In a preliminary comparison of 25 reports, the authors reported accuracy of 100% for Claude 3 Opus, 93.2% for GPT-4, and 91.5% for Gemini 1.5 Pro, with hallucination rates of 0%, 4.3%, and 5.1%, respectively [46]. These results should be interpreted as proof-of-concept findings from a controlled dataset rather than evidence of error-free performance in routine practice. The authors identified broader international validation and periodic revalidation as priorities.

Subsequent studies reinforce the need to evaluate each model within its intended laboratory task. Yang et al. examined LLMs with chain-of-thought reasoning for clinical laboratory case interpretation [47]. Nanua et al. developed a retrieval-augmented generation (RAG) system grounded in Title 42 of the Code of Federal Regulations (42 CFR Part 493) for laboratory regulatory questions [48], demonstrating how an LLM can be anchored to controlled source documents. Wang et al. developed the Laboratory-AI framework for RAG-supported personalized laboratory-test interpretation and reported improved retrieval of relevant interpretative factors and normal ranges compared with corresponding non-RAG approaches [49].

RAG is particularly attractive for laboratory medicine because a controlled knowledge base can contain validated test directories, local reference intervals, specimen requirements, analytical interferences, standard operating procedures, professional guidelines, and regulatory documents. Its quality depends on the accuracy and versioning of the retrieved sources, making source provenance and document control essential components of laboratory implementation.

Evaluation using authentic reports is increasingly important. Liu et al. compared five contemporary LLMs using 120 real-world urinalysis reports from patients with kidney disease [50]. Laboratory technologists and physicians evaluated the generated interpretations. Although several models performed well overall, analyzer-specific information and hallucinations remained limitations. Use of complete reports rather than isolated questions exposed difficulties associated with instrumentation and report structure.

LLMs have also been evaluated for laboratory terminology standardization. Yu et al. tested ChatGPT-4.0, Gemini, and Perplexity for mapping 75 laboratory tests, 55 from clinical chemistry to Logical Observation Identifiers Names and Codes (LOINC). Complete-match performance varied substantially among models, and method-related differences accounted for many partial matches [51]. These findings suggest that LLMs may generate candidate mappings, but local standardization and expert validation remain necessary.

Multimodal LLMs provide another emerging application. Yang et al. evaluated five multimodal models using 177 proficiency-testing images from blood smears, urinalysis, and parasitology [52]. Although blood-smear and parasitology interpretation extend beyond core clinical chemistry, the study illustrates transferable issues in multimodal model validation. Performance depended substantially on prompting strategy; with zero-shot prompting, the best-performing model achieved 78.5% overall accuracy and 92.0% accuracy for urinalysis images.

Bhasuran et al. evaluated causal reasoning of LLMs using 99 contextualized laboratory-test scenarios involving HbA1c, creatinine, vitamin D, and relevant clinical factors [53]. Performance declined as the reasoning task became more demanding, particularly for counterfactual reasoning. Qi et al. recently compared ChatGPT-4o, Gemini-2.5, Grok-3, and DeepSeek-R1 using 200 tumor-marker reports [54]. The models showed meaningful differences in interpretative scores and hallucination rates.

These studies suggest several potential roles for LLMs in the postanalytical phase: drafting patient-friendly explanations, supporting interpretative comments, answering laboratory-specific questions, assisting test selection, retrieving controlled knowledge, and prioritizing reports requiring specialist attention. These applications nevertheless require task-specific validation before routine implementation.

## 8. Challenges Specific to LLMs

LLMs differ fundamentally from conventional ML systems commonly used in laboratory medicine. Traditional ML models generally operate on structured numerical or image-based inputs and generate predefined outputs such as classifications, probabilities, or predicted values. In contrast, LLMs generate natural-language responses probabilistically and may produce different outputs depending on the prompt, model configuration, and contextual information provided. These characteristics introduce additional challenges related to reliability, reproducibility, transparency, governance, and patient safety [39,40,41,47,48,49,50,51,52,53,54]. Such concerns are particularly relevant in clinical chemistry, where generated interpretations or recommendations may influence diagnostic and therapeutic decisions.

### 8.1. Hallucinations

Hallucination is one of the most widely recognized limitations of LLMs and refers to the generation of information that appears plausible but is factually incorrect, unsupported by the available evidence, or fabricated [40,41]. Hallucinations may include incorrect interpretations of test results, inaccurate reference information, inappropriate recommendations, or statements that are inconsistent with the analytical or clinical context. Because laboratory reports often contain specialized terminology and method- or instrument-dependent information, even minor inaccuracies may have important consequences for interpretation.

Reported hallucination rates vary according to model, task, and implementation strategy [46,50,54]. Hallucination risk should therefore be evaluated for each intended laboratory application rather than assumed to be a fixed characteristic of a given model. Outputs should be verified against source laboratory data and other authoritative information, with professional review maintained where generated content may influence clinical interpretation or patient management.

### 8.2. Prompt Dependence and Reproducibility

LLM outputs are influenced by the wording, structure, and amount of contextual information contained in the prompt [47,52]. Changes in prompt formulation can alter the emphasis, completeness, and accuracy of generated responses even when the underlying laboratory information is unchanged. This sensitivity has been demonstrated in multimodal and laboratory case-interpretation studies, in which performance varied across prompting strategies [42,47,50].

Reproducibility may also be affected by model-generation settings such as temperature, sampling strategy, and other decoding parameters, which are distinct from prompt-related variability. For laboratory applications, both the prompt structure and relevant generation settings should therefore be standardized and documented as part of the validated workflow. Changes in the underlying model or model version represent an additional source of variability and are considered separately in the following section.

### 8.3. Model Drift and Version Instability

LLM performance may change over time for reasons arising both from the model itself and from the local environment in which it is used. Commercial LLM providers may update model architecture, training procedures, safety mechanisms, or other system components, potentially altering responses to identical inputs across model versions [5,63,74]. For this reason, the specific model and version used in a validated laboratory workflow should be documented, and substantive model updates should trigger reassessment of performance.

A separate source of performance change arises from shifts in the local laboratory environment. Changes in analyzers, analytical methods, reference intervals, reporting formats, patient populations, or clinical workflows may alter the characteristics of the data presented to the model and reduce the validity of previous evaluations. These local changes should be distinguished from vendor-driven model updates because they may require different monitoring and revalidation strategies. Continued performance monitoring and periodic revalidation are therefore important components of LLM governance in laboratory practice [5,74,75].

### 8.4. Explainability and Transparency

Explainability remains an important challenge for LLM-based applications in laboratory medicine. Unlike conventional rule-based systems, in which decision criteria can often be explicitly reviewed, modern LLMs rely on highly complex neural-network architectures whose internal processing cannot be readily interpreted [39,40]. Although LLMs can generate textual explanations for their outputs, these explanations should not be assumed to represent the actual computational processes that produced the response.

This limitation is particularly relevant in clinical laboratories, where traceability, documentation, and auditability are central components of quality management. Li and Plebani emphasized that AI-based laboratory systems require not only metrological traceability of analytical measurements but also appropriate documentation of algorithm development, training data, preprocessing procedures, model versions, and subsequent updates [76]. For commercially developed LLMs, however, full transparency may be difficult to achieve because details of model architecture, training data, and internal updates are often not publicly available.

### 8.5. Data Privacy and Confidentiality

Data privacy is a major consideration when patient information is processed by LLM-based systems. Laboratory reports frequently contain sensitive health information and personal data that may be subject to institutional and regulatory requirements. The use of externally hosted or cloud-based LLM services may create additional concerns regarding data transmission, storage, access, and secondary use [39,40].

De-identification can reduce privacy risk, but laboratory data may remain potentially identifiable when combined with demographic, clinical, or longitudinal information. Clinical institutions should therefore evaluate data governance policies, cybersecurity safeguards, access controls, data retention practices, and contractual arrangements before integrating external LLM services into laboratory workflows [5,74].

RAG may improve factual grounding by restricting responses to validated local documents or controlled knowledge sources [48,49], but it does not by itself eliminate privacy risk. If patient-specific data are transmitted to an external model, confidentiality concerns remain regardless of whether the retrieved knowledge base is locally controlled. Privacy protection therefore depends on the overall system architecture and data flow, not solely on the use of RAG.

### 8.6. Liability and Professional Accountability

The use of LLMs in laboratory workflows also raises questions regarding professional responsibility and accountability. When an LLM generates an inaccurate or inappropriate interpretation, responsibility may involve several parties, including the model developer, software vendor, healthcare institution, and laboratory professionals involved in reviewing or acting on the output. The applicable responsibilities may vary according to the intended use of the system, the degree of automation, and the extent to which the generated output directly influences patient care [5,8,74].

These issues are particularly important for patient-facing interpretation, clinical decision support, and automatically generated laboratory comments. When an LLM contributes to report generation or interpretation, qualified laboratory professionals should retain responsibility for reviewing the appropriateness and accuracy of the final output before it influences patient management. Current professional recommendations therefore support a human-in-the-loop approach, particularly for applications with direct clinical consequences [2,5,8].

Collectively, these limitations support treating current LLMs as assistive tools requiring structured validation and professional oversight [5,8,40,46,54,74].

Supplementary Table S1 summarizes the characteristics of representative primary studies discussed in this review, including study design, sample size, AI model, comparator, validation strategy, performance measures, evidence of real-world implementation, and key limitations. The included studies span different stages of evidence, from proof-of-concept and retrospective development to external validation and, less commonly, operational evaluation in routine laboratory settings.

## 9. Critical Appraisal of Current Evidence

As summarized in Supplementary Table S1, the evidence supporting AI applications in laboratory medicine spans different stages of development, validation, and implementation. Despite the breadth of these applications, the current evidence base has several methodological and translational limitations that should be considered before widespread implementation. These limitations include the predominance of retrospective studies, limited external validation, challenges associated with rare-event detection, potential publication bias, and a relative scarcity of implementation-focused investigations. These limitations are discussed below.

### 9.1. Predominance of Retrospective Validation

Among the primary studies considered in this review, retrospective study designs are common, particularly for applications based on LIS data, electronic health records, or archived digital images [12,13,14,15,16,17,19,20,21,22,23,24,27,28,29,30,31,32,33,34,38]. Retrospective datasets provide a practical basis for model development and initial validation because large numbers of observations can be analyzed without directly affecting patient care. This approach has supported the development of ML models for specimen misidentification, PBRTQC, electrophoretic interpretation, urinalysis, and autoverification [13,14,15,16,17,18,19,20,21,27,28,29,30,31,32,33,34,38,69].

However, retrospective validation has serious limitations. Historical datasets are collected under conditions that may differ substantially from those encountered after the development phase. Factors such as user interactions and unforeseen operational challenges cannot be adequately assessed retrospectively [6,74]. Furthermore, retrospective studies often assume that historical labels and classifications in supervised learning represent a definitive reference standard, which is not the case; in practice, some laboratory errors, contamination events, and interpretation discrepancies may occur. As a result, retrospective performance estimates may overstate later real-world effectiveness. Thus, prospective evaluation is required, particularly for AI systems ultimately intended to influence diagnostic or clinical decisions [8,75].

Prospective silent deployment, in which ML algorithms operate side-by-side with routine laboratory processes without influencing result release, may provide a critical intermediate step between retrospective validation and active clinical deployment [5,75]. Such real-time evaluations allow assessment of operational performance under real-world laboratory conditions while maintaining patient safety.

### 9.2. Limited External Validation and Generalizability

Among the studies considered in this review, a substantial proportion were developed and validated within a single institution, creating uncertainty regarding transportability to other laboratory environments [6,8,59]. Clinical chemistry data are influenced by laboratory-specific factors including analytical platforms, reagent systems, calibration procedures, population characteristics, measurement units, reference intervals, and reporting practices [5,7,35,60,61].

Several recent studies have incorporated multicenter datasets or independent external validation, including investigations of PBRTQC [27], sample misidentification detection [21], and laboratory-test utilization [36]. However, validation across heterogeneous analytical systems and healthcare settings remains less common than internal evaluation. Performance achieved on one analytical platform or patient population may therefore not be maintained after transfer to another laboratory environment.

Transportability is also influenced by differences in patient demographics, disease prevalence, referral patterns, and healthcare utilization [5,27,74]. AI models may therefore learn laboratory-specific or population-specific patterns that do not generalize beyond the development setting. These considerations support the need for external validation and local verification before implementation in a new laboratory environment [8,76].

### 9.3. Rare-Event Detection and Low-Prevalence Outcomes

Several AI applications considered in this review address rare but clinically important events, including WBIT errors, specimen contamination, and other preanalytical failures [19,20,21,22,23,24,25,26]. These applications present a particular evaluation challenge because low event prevalence can make conventional summary metrics appear favorable even when the number of false-positive alerts is operationally important.

For rare outcomes, small reductions in specificity may generate a substantial number of false alerts requiring manual investigation, while false-negative events may carry important patient-safety consequences despite having little effect on overall accuracy or AUROC. Studies of WBIT detection illustrate this problem clearly [22,23,24].

Evaluation of rare-event detection systems should therefore include measures that reflect routine laboratory use, such as PPV, false-alert rate, false-negative rate, alerts generated per number of specimens tested, and the resulting manual-review workload, where these data are available [22,23,24]. Similar considerations apply to contamination-detection and multivariate anomaly-detection systems [26,58]. Where such operational measures are not reported, this should be recognized as a limitation when interpreting apparent model performance.

### 9.4. Publication Bias and Reporting Limitations

Interpretation of the current evidence should also consider the possibility of publication bias. Studies reporting favorable model performance may be more likely to be submitted, published, and cited than studies with negative, neutral, or inconclusive findings. As a result, the published literature may overrepresent successful applications and provide an overly optimistic impression of implementation readiness.

This concern may be particularly relevant to commercial systems, proprietary algorithms, and locally developed models for which unsuccessful evaluations may remain unpublished. In addition, many studies emphasize discrimination metrics while providing limited information on implementation barriers, workflow effects, maintenance requirements, or long-term performance [5,6,74].

Reporting practices also vary considerably across studies, including differences in outcome definitions, validation strategies, comparator selection, and performance metrics [8]. The recent European Federation of Clinical Chemistry and Laboratory Medicine (EFLM) checklist for AI and ML-based studies in laboratory medicine provides a useful framework for improving transparency, reproducibility, and consistency of reporting [8]. Wider adoption of such reporting standards should facilitate more balanced comparison of both favorable and unfavorable findings.

### 9.5. Limited Real-World Implementation Studies

A major limitation of the evidence considered in this review is the relative scarcity of studies evaluating AI under routine operational conditions. Much of the literature focuses on model development, retrospective validation, or technical performance, whereas fewer studies assess whether implementation improves laboratory workflow, efficiency, safety, or patient care.

Metrics such as AUROC, sensitivity, specificity, and F1 score are important for characterizing model performance but do not by themselves establish operational benefit. Implementation studies should therefore also consider outcomes relevant to laboratory practice, including turnaround time, manual-review burden, false-alert workload, user acceptance, cost implications, and patient safety [5,6,74].

Evidence demonstrating sustained benefit after routine deployment remains limited across many laboratory applications. Prospective studies, including silent evaluation before active use, are therefore important for determining whether performance observed during development is maintained under real-world conditions and whether the system provides measurable workflow or clinical benefit.

## 10. Governance, Regulation, and Lifecycle Management of AI Systems

**Figure 3:**
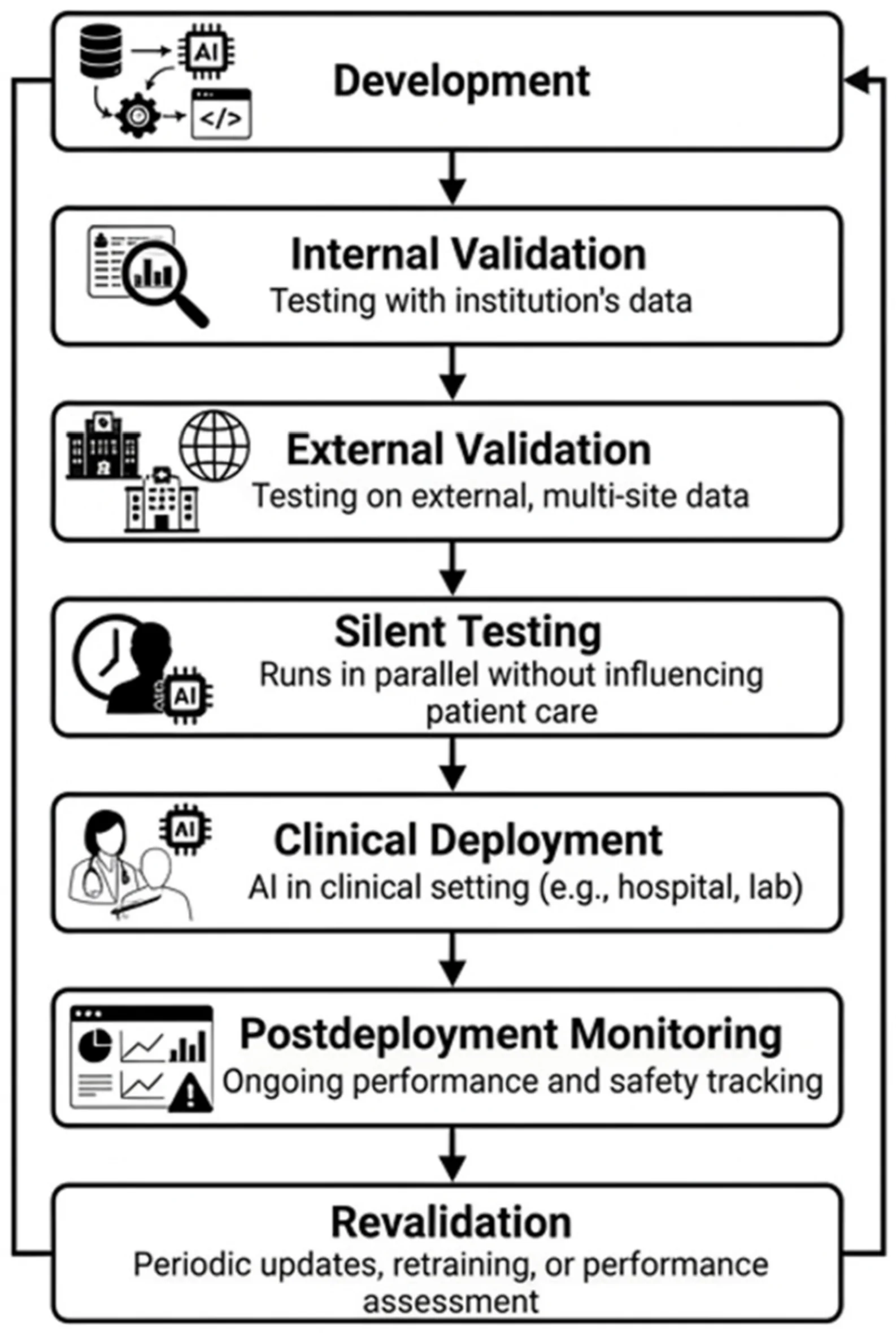
Lifecycle of AI systems in clinical chemistry. Proposed lifecycle illustrating development, internal and external validation, prospective silent testing, clinical deployment, post-deployment monitoring, and periodic revalidation of AI systems.

Implementation of AI-based systems in clinical laboratories should be approached as a continuous lifecycle encompassing development, validation, deployment, performance monitoring, change control, and periodic revalidation [8,74,75,76]. The principal stages of this lifecycle are summarized in Figure 3. This approach is particularly important because model performance may change after implementation as a result of software updates, changes in laboratory data or analytical systems, and shifts in the population or workflow in which the model is used.

The lifecycle of an AI system in the clinical laboratory extends from model development and validation to prospective evaluation, deployment, and continued performance monitoring. Internal validation establishes whether performance is maintained beyond the data used for model training, whereas external validation assesses transportability across independent populations, laboratories, or analytical environments. When these evaluations are satisfactory for the intended use, prospective silent testing can provide evidence of model behavior under routine operating conditions without affecting result release or patient management. This stage is also useful for identifying operational issues, including false alerts, workflow burden, and the level of professional oversight required. Clinical deployment should therefore be based not only on predictive performance but also on whether the system performs reliably within the intended laboratory setting. After implementation, continued monitoring is needed because model performance may be affected by software updates, analytical changes, workflow modifications, or shifts in the tested population. Revalidation may be required when such changes are substantial enough to affect the assumptions or conditions under which the system was originally evaluated [5,6,8,74,75].

Regulatory requirements depend on the intended purpose and function of an AI system rather than on the use of AI itself. The International Medical Device Regulators Forum (IMDRF) defines Software as a Medical Device (SaMD) as software intended for one or more medical purposes that performs those purposes without being part of a hardware medical device [77]. Accordingly, an AI application used for administrative support, research, or locally supervised workflow assistance does not necessarily have the same regulatory status as software intended to provide diagnostic information or directly influence patient management. The regulatory classification of each system should therefore be determined according to its intended use and the applicable regulatory framework [77].

Within the European Union, AI-based laboratory software may be regulated under Regulation (EU) 2017/746 on in vitro diagnostic medical devices (IVDR) when it meets the definition of an in vitro diagnostic medical device [78]. The regulation also includes specific provisions for devices developed and used within health institutions. In parallel, the EU Artificial Intelligence Act applies a risk-based framework to AI systems and may classify certain AI systems associated with regulated medical devices as high risk [79]. Regulatory requirements therefore depend on the intended purpose and use of the system rather than on the use of AI alone.

In the United States, the regulatory status of AI-based laboratory software depends on its intended use and function. The U.S. Food and Drug Administration (FDA) distinguishes between certain clinical decision support functions that are not regulated as medical devices and software functions that meet the definition of a medical device [80]. For AI-enabled devices, FDA guidance provides recommendations for predetermined change control plans covering planned modifications; the methods used to develop, validate, and implement those modifications; and assessment of their impact [81].

ISO 15189:2022 provides a broader quality framework for medical laboratories [82]. Although it is not specific to AI, its requirements for validation, documentation, traceability, defined responsibilities, and ongoing quality assurance are directly relevant when AI systems are incorporated into laboratory workflows.

AI-integrated nanobiosensing illustrates the additional governance challenges that arise when computational models are incorporated directly into emerging analytical platforms. Translation from proof-of-concept devices to routine clinical use requires reproducible nanomaterial fabrication, controlled surface functionalization, lot-to-lot consistency, stability in clinically relevant matrices, standardized analytical procedures, and compatibility with laboratory instrumentation and information systems [10,11]. Changes in either the sensing component or the computational model may alter final performance; consequently, change control and performance monitoring should address both components of the analytical system.

Across these different applications, governance should be proportionate to the intended use and potential consequences of failure. Relevant considerations include the degree of automation, extent of human oversight, probability and detectability of error, severity and reversibility of potential consequences, data provenance, analytical traceability, cybersecurity, auditability, version control, and post-deployment monitoring [8,74,75,76,77,78,79,80,81,82]. These factors provide a more appropriate basis for determining validation and oversight requirements than assigning risk solely according to the type of AI application.

Based on these considerations, Table 1 presents a practical framework for the risk-based evaluation of AI applications in clinical chemistry. The proposed approach considers the intended use, degree of automation, human oversight, likelihood and detectability of failure, and potential consequences of error when determining appropriate validation and monitoring requirements.

## 11. Conclusions

AI is increasingly being evaluated across the total testing process in clinical chemistry, from test selection and specimen assessment to analytical quality control, result verification, and laboratory-report interpretation. Among the studies considered in this review, comparatively stronger evidence was found for well-defined laboratory tasks with structured inputs and measurable outcomes, including specimen-error detection, patient-based real-time quality control, electrophoretic pattern recognition, selected urinalysis applications, and biochemical result verification. These applications illustrate the potential for AI to complement established laboratory processes rather than replace professional judgment.

Within the evidence considered in this review, translation into routine practice remains limited by the predominance of retrospective studies and incomplete external validation, and the influence of laboratory-specific analytical conditions. Model performance may change across patient populations, instruments, assays, calibration systems, reference intervals, and reporting environments. For rare outcomes such as specimen misidentification or contamination, conventional discrimination metrics alone are insufficient; evaluation should also consider positive predictive value, false-alert burden, false-negative events, and the number of specimens requiring manual review. Prospective assessment, including silent deployment before active use, can help determine whether technical performance is maintained under routine laboratory conditions.

LLMs extend laboratory AI beyond structured prediction toward natural-language interpretation and knowledge support, but they introduce additional concerns related to hallucinations, prompt dependence, model-version instability, explainability, privacy, and professional accountability. The evidence considered in this review supports their current use primarily as assistive rather than autonomous systems, with controlled workflows, task-specific validation, and continued review by qualified laboratory professionals.

Overall, safe implementation of AI in clinical chemistry requires a lifecycle approach that links development, internal and external validation, prospective evaluation, clinical deployment, post-deployment monitoring, and revalidation. The level of validation and oversight should reflect the intended use and the potential consequences of error. Future progress will depend less on demonstrating high algorithmic performance in isolated datasets and more on establishing transportability, operational benefit, analytical robustness, and sustained performance in real laboratory environments.

## Figures and Tables

**Table 1:** Proposed risk-based validation framework for artificial intelligence applications in clinical chemistry.

Risk Factor *	Lower-Risk Context ^†^	Higher-Risk Context ^†^	Implications for Validation and Oversight ^‡^
Intended use	Administrative, prioritization, or advisory function	Direct contribution to result reporting, diagnosis, or patient management	Validation should be appropriate to the intended clinical function and consequences of incorrect output
Degree of autonomy	AI provides information or recommendations without initiating an action	AI automatically initiates, modifies, rejects, or releases a laboratory result or action	Increasing autonomy requires stronger prospective evaluation and safeguards
Human oversight	Output is reviewed by qualified laboratory personnel before use	Output may influence care without routine professional review	Reduced human oversight increases the importance of predefined performance limits and fail-safe mechanisms
Likelihood of failure	Low error frequency under representative operating conditions	Errors are frequent, unpredictable, or sensitive to changes in input data	Validation should characterize failure frequency under representative laboratory conditions
Severity of consequences	Errors have limited operational or clinical consequences	Errors may result in incorrect reporting, delayed diagnosis, or inappropriate clinical management	Greater potential harm requires more rigorous validation and monitoring
Detectability of failure	Errors are readily recognized by users or existing quality-control procedures	Errors may appear plausible and remain undetected	Poorly detectable failures require additional controls and independent review mechanisms
Reversibility	Incorrect output can be corrected before affecting patient care	Consequences may be difficult to reverse once an output has been released or acted upon	Less reversible consequences require stronger preventive controls
Deployment environment	Use remains within the laboratory, population, workflow, and analytical system in which the model was validated	Use extends to different laboratories, populations, instruments, methods, or workflows	External validation and local verification become increasingly important as the deployment environment differs from the development setting
System changes over time	Stable model, analytical system, inputs, and workflow	Model updates or changes in analytical methods, data distributions, or workflow	Change control, ongoing monitoring, and revalidation should be incorporated into lifecycle management

* Risk depends on the intended use and implementation of the AI system rather than on the application category alone. The factors presented here provide general considerations for laboratory AI; established regulatory frameworks, including IMDRF SaMD risk categorization [83], should be applied where relevant. ^†^ The lower- and higher-risk categories are descriptive and do not represent formal regulatory classifications. ^‡^ More extensive validation and monitoring may be needed when systems operate with greater autonomy, limited human review, less detectable errors, or in a different laboratory setting.

## Data Availability

No new data were created or analyzed in this study. Data sharing is not applicable to this article.

## Supplementary Materials

The following supporting information can be downloaded at: https://www.mdpi.com/article/10.3390/molecules31203617/s1, Table S1: Characteristics, validation strategies, performance, and implementation status of representative primary studies of artificial intelligence applications in clinical chemistry and laboratory medicine. References cited in Table S1 correspond to the numbered references in the main manuscript.

## Abbreviations

The following abbreviations are used in this manuscript:

AI	Artificial intelligence
AUROC	Area under the receiver operating characteristic curve
CBC	Complete blood count
EFLM	European Federation of Clinical Chemistry and Laboratory Medicine
EU	European Union
FDA	U.S. Food and Drug Administration
IFE	Immunofixation electrophoresis
IMDRF	International Medical Device Regulators Forum
IV	Intravenous
LIS	Laboratory information system
LLM	Large language model
LOINC	Logical Observation Identifiers Names and Codes
ML	Machine learning
PBRTQC	Patient-based real-time quality control
PPV	Positive predictive value
RAG	Retrieval-augmented generation
SaMD	Software as a Medical Device
SERS	Surface-enhanced Raman spectroscopy
SPE	Serum protein electrophoresis
UTI	Urinary tract infection
WBIT	Wrong-blood-in-tube
XGBoost	Extreme gradient boosting
