# Supplementary material for: From Algorithm Performance to Laboratory Deployment: A Critical Review of Artificial Intelligence in Clinical Chemistry
Source: Molecules. 2026 Oct 11;31(20):3617. doi: 10.3390/molecules31203617 (PMC13707439; doi:10.3390/molecules31203617)
Supplement: Supplementary file 1 [file molecules-31-03617-s001.zip › molecules-4583477-supplementary.pdf]

SUPPLEMENTARY MATERIAL

From Algorithm Performance to Laboratory Deployment: A Critical Review of Artificial Intelligence in Clinical Chemistry

Abdelraheem Baniahmad [1,*] and Suleiman Bani-Ahmad [2]

[1] Department of Medical Analysis, Faculty of Applied Medical Sciences, Al al-Bayt University, Mafraq 25113, Jordan
[2] Department of Intelligent Systems, Faculty of Artificial Intelligence, Al-Balqa Applied University, Salt, Jordan; sulieman@bau.edu.jo
* Correspondence: raheem@aabu.edu.jo

CONTENTS

Table S1. Characteristics, validation strategies, performance, and implementation status of representative primary studies of artificial intelligence applications in clinical chemistry and laboratory medicine. References cited in Table S1 correspond to the numbered references in the main manuscript

Table S1. Characteristics, validation strategies, performance, and implementation status of representative primary studies of artificial intelligence applications in clinical chemistry and laboratory medicine.

Application	Study†	Sample size / design	AI model	Validation	Performance / findings	Real-world implementation / key limitation
Serum quality assessment	**Chen et al. [12]**	21,000 serum samples for development/validation plus an independent test set of 21,000; single-laboratory evaluation under real-world laboratory conditions	Two-step YOLOv5-ResNet-50 deep-learning model; compared with serum-index testing and manual assessment	Internal validation + independent test set	Recognition accuracy was 97% for hemolysis, 93% for icterus, and 97% for lipemia; exact human–machine agreement was 72% versus 42–68% for manual assessment	Prototype integrated with a Roche Cobas c 701 preprocessing module; routine deployment not established; laboratory-specific model.
Sample misidentification	**Seok et al. [19]**	246,261 tumor-marker records; retrospective study with simulated misidentification	Random forest and deep neural network versus conventional delta percent change, absolute delta percent change, and reference-change-value methods	Internal validation	ML-based delta checks provided better discrimination than conventional approaches across the evaluated tumor markers	No real-world deployment; misidentification was simulated and external validation was not performed.
Sample misidentification	**Seok et al. [21]**	397,751 results for development/internal validation plus 215,339 results from three independent laboratories; retrospective multicenter study	Deep neural networks and XGBoost versus conventional delta-check methods	Internal + multicenter external validation	ML AUROC 0.834-0.903 versus 0.705-0.816 for conventional methods; external balanced accuracy 0.760-0.836 versus 0.670-0.773	No active deployment reported; errors were simulated and performance varied among external datasets.
WBIT detection	**Tortosa-Carreres et al. [24]**	Internal cohort: n=432 (training n=324; internal validation n=108); external validation cohorts: n=800 and n=460; retrospective multicenter study	Elastic-Net feature selection + XGBoost versus conventional multivariate/delta-check approaches	Internal + external validation	High discriminatory performance was maintained across external cohorts, supporting transportability beyond the development site	No prospective routine deployment; rare-event prevalence and threshold selection remain important limitations.

Application	Study†	Sample size / design	AI model	Validation	Performance / findings	Real-world implementation / key limitation
PBRTQC	**Liang et al. [27]**	Training data comprised 6,786,110 hematology and 3,097,661 chemistry data points from eight hospitals; external validation in three independent hospitals; multicenter retrospective study	ML nonlinear regression-adjusted PBRTQC versus IFCC PBRTQC and linear regression-adjusted PBRTQC	Internal + multicenter external validation	mNL-PBRTQC outperformed both comparators for all evaluated measurands and simulated biases in the three independent datasets	No routine deployment reported; analytical errors were introduced by simulation.
PBRTQC	**Dong et al. [28]**	Five months of routine patient data for six biochemical/immunological analytes; data volume ranged from 734 to 11,343 results per analyte in the AI-PBRTQC group; single-laboratory operational evaluation	AI-supported PBRTQC versus traditional PBRTQC	Operational evaluation	AI-PBRTQC more efficiently identified several quality risks and detected events associated with calibration and reagent-related changes	Evaluated using real laboratory quality events; single-center setting and performance varied by analyte.
IFE pattern recognition	**Hu et al. [13]**	>12,000 expert-annotated IFE images; retrospective image-based study using images from different acquisition systems	Ensemble deep-learning model versus laboratory experts	Internal/cross-system validation	Expert-level recognition of major immunoglobulin and light-chain patterns; explainability maps identified image regions contributing to classifications	No routine workflow implementation demonstrated; retrospective image-based evaluation.
SPE M-protein detection	**Elfert et al. [14]**	69,722 individuals; retrospective study with expert-comparison test set	Five ML methods; random forest performed best; compared with laboratory experts	Internal validation	Random forest achieved 99.1% accuracy, 89.9% sensitivity, 99.8% specificity, and F1 score 93.2%	No external prospective implementation; performance derived from a retrospective dataset.
SPE interpretation	**Lahtiharju et al. [15]**	34,625 Finnish patients; retrospective validation	Neural-network models for fractionation, classification, and paraprotein integration	Retrospective validation	Classification AUROC approximately 0.97; supported feasibility of partial automation of SPE interpretation	No prospective workflow deployment; single-population retrospective evaluation.

Application	Study†	Sample size / design	AI model	Validation	Performance / findings	Real-world implementation / key limitation
UTI prediction from urinalysis	**Favresse et al. [69]**	22,961 samples; retrospective cohort with independent test set	Six ML models incorporating demographic and dipstick-reflectance data; CatBoost among best performers	Internal development + independent test set	AUROC approximately 0.92-0.95 depending on UTI definition; negative predictive value remained high	No prospective implementation; effect on urine-culture workload or prescribing not established.
SERS-based biomarker classification	**Dixon et al. [70]**	Up to 300 unique simulated serum samples containing randomized combinations of five cardiovascular biomarkers; analytical proof-of-concept	Ag-nanoparticle SERS + ML classification	Internal experimental validation	Demonstrated classification of multidimensional spectral signals; model performance was strongly influenced by experimental and acquisition conditions	No clinical deployment; simulated samples and platform/batch variability limit generalizability.
ML-assisted nanobiosensor	**Wang et al. [71]**	NR	Gold-nanoparticle lateral-flow assay combined with ML	Analytical/translational validation	Demonstrated highly sensitive quantitative detection of tau with ML-assisted signal interpretation	Translational rather than routine clinical implementation; external clinical validation and inter-platform reproducibility remain needed.
SERS classification of colorectal lesions	**Zhu et al. [72]**	100 clinical serum samples: 30 healthy controls, 30 patients with colorectal precancerous lesions, and 40 patients with colorectal cancer	Au/SnO2 SERS + PCA-OCDCO ML model	Internal clinical validation	Accuracy 97%, sensitivity 95%, specificity 97%, AUC 0.96	No external validation or routine deployment; batch and platform reproducibility remain relevant.
Biochemistry report verification / autoverification	**Zhu et al. [38]**	6,382 laboratory reports containing 38 biochemical variables plus demographic information; retrospective development with double-blind testing	Random forest, XGBoost, and logistic regression	Cross-validation + operational testing	Random forest AUROC 0.985; pass rate 88.14%; false-negative rate 0.62%; median turnaround time decreased from 89 to 68 min	Operational benefit was evaluated, but multicenter external validation was not reported.

Application	Study[†]	Sample size / design	AI model	Validation	Performance / findings	Real-world implementation / key limitation
LLM laboratory-report interpretation	**D'Urso et al. [46]**	100 laboratory reports from three Italian laboratories; controlled evaluation by laboratory-medicine experts	Claude-based domain-restricted system; preliminary comparison with GPT-4 and Gemini	Controlled multicenter report evaluation	Reported high technical interpretation accuracy with no hallucinations in the controlled primary evaluation; patient feedback was predominantly positive	Controlled proof-of-concept rather than routine deployment; small dataset and highly constrained prompting/workflow.
LLM interpretation of urinalysis reports	**Liu et al. [50]**	120 real-world urinalysis reports from patients with kidney disease; two analyzer platforms	Five contemporary LLMs evaluated by laboratory technologists and physicians	Retrospective real-world report validation	Several models showed useful interpretative performance, but analyzer-specific errors and hallucinations persisted	No active deployment; small retrospective dataset and strong model/version dependence.
LLM-assisted LOINC mapping	**Yu et al. [51]**	75 laboratory tests, including 55 clinical chemistry and 20 hematology tests	ChatGPT-4.0, Gemini 1.5, and Perplexity AI versus consensus mapping by six clinical pathologists	Controlled benchmark validation	ChatGPT-4.0 achieved the highest complete-match rate in clinical chemistry (58.2%); method-related discrepancies accounted for many partial matches	No deployment; expert verification remains necessary for local standardization.
Multimodal laboratory-image interpretation	**Yang et al. [52]**	177 proficiency-testing images: 78 blood smears, 50 urinalysis, and 49 parasitology images	Five multimodal LLMs tested with three prompting strategies	Controlled comparative validation	Best zero-shot model achieved 78.5% overall accuracy and 92.0% accuracy for urinalysis images; performance varied substantially by prompting strategy	No clinical deployment; performance differed markedly across image domains and prompts.
LLM causal reasoning for laboratory interpretation	**Bhasuran et al. [53]**	99 clinically grounded laboratory-test scenarios	GPT-o1 versus Llama-3.2-8B-Instruct; evaluated by four medically trained experts	Controlled benchmark validation	Overall AUROC 0.80 versus 0.73; GPT-o1 also showed higher sensitivity and specificity; both models performed least well on counterfactual reasoning	Scenario-based evaluation rather than routine reports; no deployment evidence.

Application	Study†	Sample size / design	AI model	Validation	Performance / findings	Real-world implementation / key limitation
LLM tumor-marker report interpretation	**Qi et al. [54]**	200 tumor-marker reports; retrospective single-center study	ChatGPT-4o, Gemini-2.5, Grok-3, and DeepSeek-R1; evaluated by four laboratory technologists	Comparative retrospective validation	Mean scores ranged from 3.82 to 4.31/5; hallucination rates ranged from 1.0% to 11.5%; all models were weaker in cause analysis and clinical recommendations	No deployment; single-center study and results are strongly model/version dependent.

*Abbreviations: AI, artificial intelligence; AUC, area under the curve; AUROC, area under the receiver operating characteristic curve; DNN, deep neural network; IFE, immunofixation electrophoresis; LLM, large language model; ML, machine learning; NR, not reported; PBRTQC, patient-based real-time quality control; SERS, surface-enhanced Raman spectroscopy; SPE, serum protein electrophoresis; UTI, urinary tract infection; WBIT, wrong blood in tube; XGBoost, extreme gradient boosting.

†References cited in Table S1 correspond to the numbered references in the main manuscript
